# Nanoforging – Innovation in three-dimensional processing and shaping of nanoscaled structures

**DOI:** 10.3762/bjnano.5.118

**Published:** 2014-07-16

**Authors:** Andreas Landefeld, Joachim Rösler

**Affiliations:** 1 Institut für Werkstoffe, Technische Universität Braunschweig, Langer Kamp 8, 38106 Braunschweig, Germany

**Keywords:** forging, manipulation, nanoforging, plastic deformation, tungsten

## Abstract

**Background:** This paper describes the shaping of freestanding objects out of metallic structures in the nano- and submicron size. The technique used, called nanoforging, is very similar to the macroscopic forging process.

**Results:** With spring actuated tools produced by focused ion beam milling, controlled forging is demonstrated. With only three steps, a conical bar stock is transformed to a flat- and semicircular bent bar stock.

**Conclusion:** Compared with other forming techniques in the reduced scale, nanoforging represents a beneficial approach in forming freestanding metallic structures, due to its simplicity, and supplements other forming techniques.

## Findings

The macroscopic world offers a large variety of three-dimensional forming processes, for example casting, forging or cutting. Producing three-dimensional mechanical resilient structures in the submicron- and nanoscale is a very challenging task. Generally, when size is reduced it becomes more and more difficult to position nanoobjects and to control their geometry in three individual dimensions. Adhesive effects become dominant and forces such as gravity are negligible. Other macroscopical versatile processes like casting or cutting are not reproducible to the nanoscale, at the moment. For example, studies on chip formation in the nanoscale are a wide field of research at present [1–2]. But three dimensional cutting in the nanoscale is still unexplored. Casting is also limited in its nano-applicability. The mold filling depends on the mold temperature and the filling pressure [3] and is also limited by the surface tension of the cast-material. Some complex three dimensional shapes were realized by casting of metal with a low melting temperature [3–4]. Dimensions of cast details are in the submillimeter- and microscale, and due to the mentioned limitations, applications in the submicron- and nanoscale do not seem to be feasible. For electromechanical and optical application numerous patterning techniques were developed. Only a few are applicable for patterning metals. An example is imprinting. This techniques uses a macroscopic stamp with structures in the micro- and nano-range. By pressing it into a substrate the structure of the stamp is replicated as imprint. Polymers [5], but also metallic glasses [6], are used as substrate material for this surface patterning process. It is less applicable for three-dimensional forming of individual objects than for structuring of large and plain surfaces. Another example is electrodeposition [7]. This technique enables three dimensional patterning in the nano-range by deposition of metals electrochemically. Like imprinting, electrodeposition is less suitable for direct and individual shaping of freestanding structures.

Recently Landefeld et al. [8] and Schloesser et al. [9] showed that forging has a great potential to form miniaturized freestanding objects. In their studies cubic single crystalline Ni_3_Al-particles were compressed, investigating the true strain-true stress response of the freestanding nanoparticles. High degrees of deformation (true strain φ > 1) were observed without any signs of cracking [8,10–11]. The measured yield strengths of 2500 MPa to 5000 MPa are a significant portion of the theoretical strength [8]. Landefeld and Rösler [12] successfully produced defined forged shapes out of the single crystalline cubic Ni_3_Al-particles. They used lithographically etched dies as a mold for open die forging. Other techniques like free forming were also studied on these particles. This only shows a small part of shapes forging enables.

It turned out that it is helpful to simplify processes when scaled down. In this respect forging is an attractive forming process where complex three-dimensional structures can be realized with simple tools. In the most elementary way a bar stock is forged between two rams which move towards each other. The shaped surfaces are small in nanoforging. High forces sometimes set boundaries in macroscopic forging, which is not an issue when microscopic material volumes are formed. As mentioned above, the small volume of the used material also yields mechanical properties which are not achievable in macroscopic forging. Consequently high degrees of deformation, good mold filling as well as complex shapes can be more readily achieved. Single crystalline silicon, processed by focused ion beam milling, is used for durable spring actuated forging tools. Furthermore, high strength metals can be processed and high deformation degrees of the bar stock can be realized without heating. This is notably interesting for structural components and when magnetic or electrical properties of metals are desired. Due to forging in the vacuum of a scanning electron microscope (SEM) the unwanted oxidation can be minimized.

**Figure 1:**
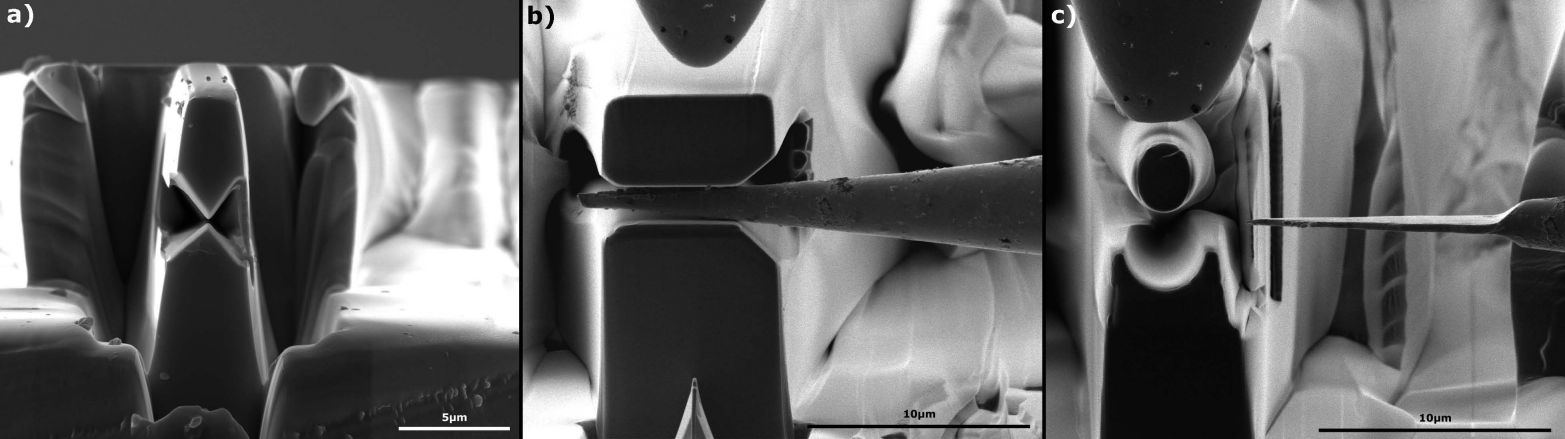
Scanning electron microscope images of different forging tools. Image a) shows a cutting tool, b) a freeforming tool and c) a bending tool.

The experiments were done in a DualBeam microscope (FEI Helios NanoLab^TM^ 650). A system of two micromanipulators (MM3A-EM from Kleindiek Nanotechnik) was mounted on the stage and at the door of the microscope in a angle of 90° to each other. Regarding nanoforging, the positioning accuracy is 3.5 nm and 5 nm, respectively, in the two rotational axes and 0.25 nm in the linear axis [13]. The forging tools were machined by focused ion beam milling at the corner of a single crystalline Si-substrate. Varying tools in different positions were produced to allow several forging steps after each other (Figure 1).

All of these tools are based on a spring principle similar to so called spring swages which were known in historic blacksmith shops. The upper spring arm moves down when pressing straight on it, consequently the upper and the lower arm come closer, resulting in shaping of the bar stock between the forging surfaces. After load relief the upper spring arm moves in its initial position. This principle allows fast and precise forging between both forming surfaces (video Supporting Information File 1). The bar stock was produced from a 0.5 mm drawn polycrystalline tungsten wire with a purity of 99.995%. Tungsten can be relatively easy machined electrochemically. It is often used for tough and sharp probes in scanning probe microscopy. An electrochemical process described in [12] is applied here to produce the essentially conical bar stock, having a diameter of 100 to 300 nm at the tip and 1000 to 4000 nm at the forging tail.

**Figure 2:**
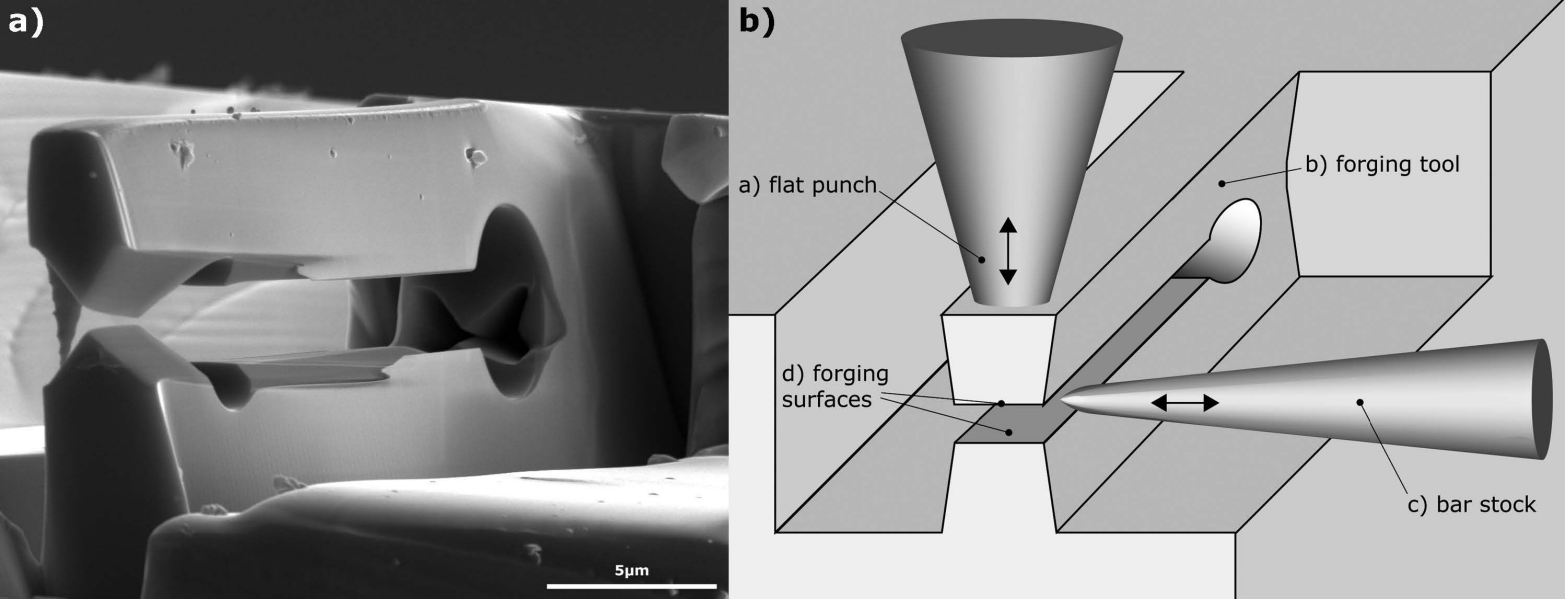
Spring principle of a cutting tool on the left (a). Installation schematically on the right (b). The upper bending arm of the forging tool moves down when pressing on it with the flat punch. It moves in its initial position while load release due to the elastic force. The bar stock is moved between the forging surfaces.

Figure 2b illustrates a possible installation where the flat punch (a) actuates the forging tool (b) and shapes the bar stock (c) between the forging surfaces (d). The forging tools can be arranged in a staggered way, so that different forging steps can be done after each other without the need of time and effort of tool changing. The geometries of the forging tools were varied to show exemplary three different possibilities of nanoforging. A cutting tool, a freeforming tool and a bending tool is shown in Figure 1. The function of the three forging tools is described below.

**Figure 3:**
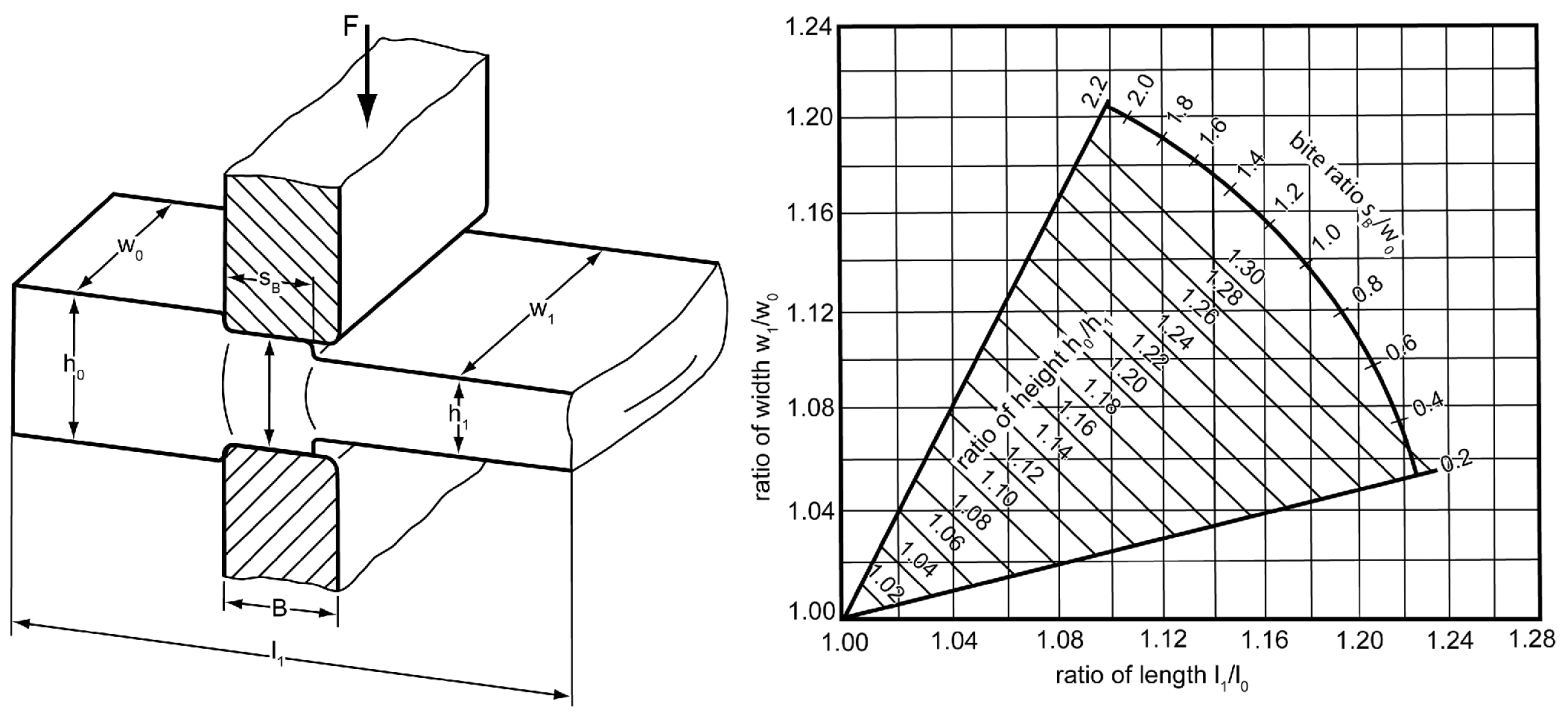
The grade of stretching and spreading is influenced by the bite ratio *s*_B_/*w*_0_. Illustrations after Spur and Stöferle [14].

**Figure 4:**
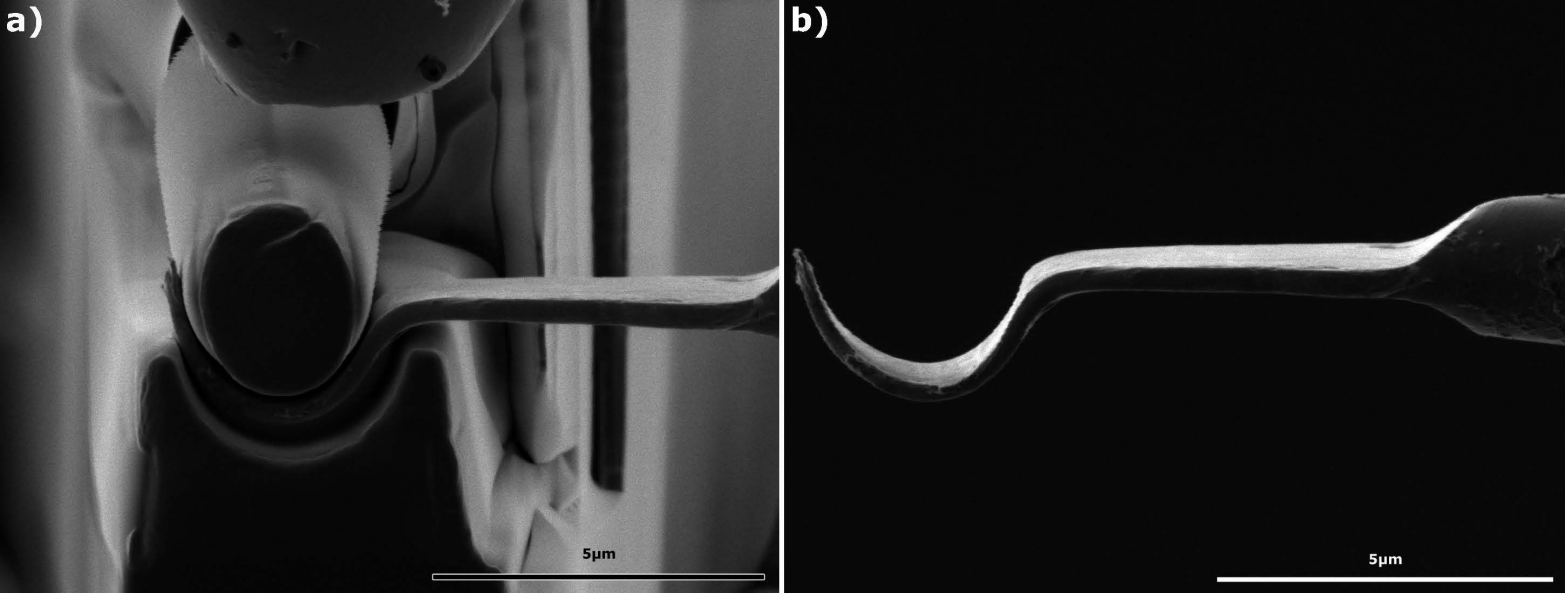
SEM images of the bending process. The flat bar is forged in the lower forging die and adapted its outer geometry. The inner shape is given by the cylindrical upper bending arm.

i) Trimming via shearing-off is used when the tip of the bar stock needs to be cut to length. In this demonstration, the forging tool is designed with two sharp symmetrical wedges (Figure 2a) and a wedge angle of 30° and 40°. The intersecting angle of the cutting surface is consequently similar to the wedge angle. The video Supporting Information File 2 shows a cut on a tungsten bar stock exemplarily.

ii) Forging between even surfaces is a free forming process also common in macroscopic forging processes. Basically there are two different kinds, the stretch forging and the spread forging. The grade of stretching and spreading can be influenced in the following ways. First the bar stock can be rotated by 90° after each forging step resulting in a stretched workpiece. Second the bite ratio *s*_B_/*w*_0_ influences the material flow. It is defined as ratio of the bite *s*_B_ and the width *w*_0_. The lower the bite ratio, the higher the grade of stretching (Figure 3) [14]. For example, when a bite ratio *s*_B_/*w*_0_ of 0.2 and a reduction of height *h*_0_/*h*_1_ by a factor of 1.21 is choosen, the bar stock is stretched by a factor of *l*_1_/*l*_0_ = 1.16 and spread by a factor of *w*_1_/*w*_0_ = 1.04. The video Supporting Information File 1 shows a tungsten bar stock flattened by forging between even surfaces. Figure 1c shows a flattened bar stock before it was bent in the next step. In this demonstration the initial thickness of 680–1800 nm of the conical bar stock was reduced to 200–300 nm. Still, there is potential to reduce the thickness below 100 nm. An correlation of the material flow between macroscopic forging and nanoforging was found. In this study the bite ratio was about 2.2, thus the bar stock was as expected more spread than stretched. However, due to the conical geometry of the bar stock, it was not possible to give an exact comparison between Figure 3 and the results of nanoforging.

iii) The third forging tool allows the bending of a flat bar to a semicircle. A semicircular forging die and a cylindrical upper spring arm defines the contour of the flat bar. The lower side is stretched and the upper side is compressed resulting in a semicircle geometry of the flat bar. Figure 4a and video Supporting Information File 3 shows how the flat bar is formed into the lower die. When lifted out it can be seen that the upper bending arm is broken, due to a too high chosen distance between the upper and the lower arm of the forging tool. Nevertheless a smooth and clear geometry is reproduced (Figure 4b).

In summary we have given an overview of the forging process in the submicron- and nanoscale. A tungsten bar stock with a taper diameter of 2 μm at the forging end was first cut, than stretched and broadened and finally bent to a semicircle. On the basis of these three techniques it is exemplary shown that forging offers a great potential in shaping of three-dimensional structures in the submicron- and nano-range. The forging tools, produced here by focused ion beam milling of a single crystalline silicon wafer, can be individually designed and adjusted to the needs. Therefore, forging parts with a great variety of shapes are possible. With little constraint much of the macroscopic forging technique is realizable in the nanoscale. Typical parts produced by forging could be axle-shafts, discs, flat or spiral springs. Furthermore, bending and cutting of nano wires could be also possible applications of nanoforging. In contrast to structures produced by ion- or electron beam induced deposition, homogenous metallic structures can be manufactured with a high mechanical load capacity. Nanoforging plays out its strength when a small quantity and flexible and individual control of shape is required as well as mechanical properties of metals are of interest.

## Supporting Information

File 1 Tungsten bar stock trimmed by a cutting tool.

File 2 Tungsten bar stock flattened by forging between even surfaces.

File 3 Tungsten flat bar bent to a semicircle geometry.
